# Hsa‐circ‐0052001 promotes gastric cancer cell proliferation and invasion via the MAPK pathway

**DOI:** 10.1002/cam4.5446

**Published:** 2022-12-01

**Authors:** Qixuan Xu, Yizhou Yao, Haishun Ni, Jinrong Gu, Xuchao Wang, Linhua Jiang, Bin Wang, Xinguo Zhu

**Affiliations:** ^1^ Department of General Surgery The First Affiliated Hospital of Soochow University Suzhou People's Republic of China

**Keywords:** circRNA, gastric cancer, hsa‐circ‐0052001, MAPK, miR‐608

## Abstract

**Background:**

Gastric cancer (GC) ranks fourth among the causes of death from malignant tumors in the world. Studies have implicated the dysregulation of circRNAs with GC. However, the relationship between hsa‐circ‐0052001 and GC is unclear.

**Methods:**

In our current study, we assessed the expression levels of hsa‐circ‐0052001 in GC cells and tissues using quantitative real‐time PCR (qPCR). The role of hsa‐circ‐0052001 expression on the proliferation and invasion of GC cells was assessed using in vitro experiments. The role of hsa‐circ‐0052001 on the proliferation of GC cells was also analyzed using in vivo models. The pathways downstream of hsa‐circ‐0052001 were identified using bioinformatics analyses, western blot (WB) assays, and qRT‐PCR.

**Results:**

We found that compared with normal gastric mucosa epithelial cells and adjacent paracancer tissues, hsa‐circ‐0052001 was overexpressed in GC cells and tissues. Also, the hsa‐circ‐0052001 level was linked to patient clinicopathological characteristics of GC. Cell proliferation and metastatic ability were inhibited in gastric cancer cells when hsa‐circ‐0052001 was knocked down in vitro and cancer growth in vivo. Mechanistically, hsa‐circ‐0052001 promoted the carcinogenesis of GC cells via the MAPK signal pathway.

**Conclusion:**

Hsa‐circ‐0052001 functions as a tumor gene in promoting the progression of GC through MAPK pathway, which has provided a promising target for patients with GC.

## Graphical abstract

This study provides important insights into the expression of hsa‐circ‐0052001 in patients with gastric cancer and its influence on the prognosis of patients. The data showed that hsa‐circ‐0052001 expression was increased in patients with gastric cancer and suggested a poor prognosis.

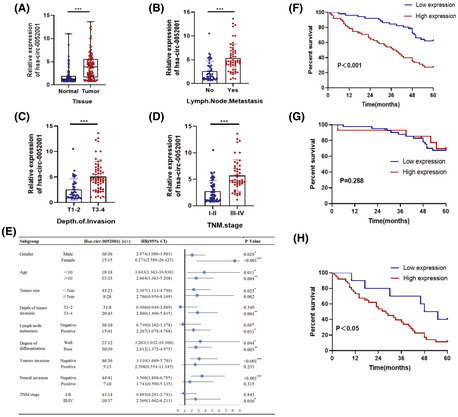

## 1 INTRODUCTION

Global cancer statistics presented in 2020 indicate that GC is the fifth malignant cancer in the world and the fourth cause of tumor‐induced mortality across the world. East Asia has the highest GC incidence, particularly in men in Japan and women in Mongolia, with Northern America and Northern Europe reporting some of the lowest GC incidences.^1^ Given the lack of clear clinical signs for early‐stage GC, cancer is usually diagnosed in a later stage.^2^ Despite the recent advances in treatment, including surgery, chemotherapy, and radiation, late‐stage gastric cancer is very difficult to treat.^3^ Therefore, there is an urgent to explore more accurate methods for early‐stage GC diagnosis. Diagnostic markers and the molecular mechanisms underlying gastric cancer can reveal novel targets for GC treatment and diagnosis. Circular RNAs (circRNAs) are a new kind of non‐coding RNAs that are featured by the existence of covalence bonds that link the 3′ and 5′ ends through back splicing.^4^, ^5^, ^6^, ^7^ Previously, they were formerly dismissed as meaningless byproducts of gene rearrangement and splicing. Recently, researches have revealed that circRNA is relatively stable, and conserved, and its expression levels vary among different tissues and cell types, due to the fast progress of novel sequence identification technologies and biological information analysis.

CircRNAs perform numerous functions, including sponging with miRNA, protein binding (RBPs), and regulating protein translation.^8^, ^9^ Recent studies have implicated certain circRNAs in gastric cancer development and progression. For example, circDONSON promoted GC cell progression and development via the activation of SOX4 signaling.^10^ Meanwhile, downregulating circRNA‐100269 suppressed GC cells proliferation and growth by interacting with miR‐630.^11^ Circ‐SFMBt2 was upregulated in GC tissues and enhanced the proliferation of GC cells.^12^ These researches imply that some circRNAs might play an important role in the progression of GC. However, more circRNAs in GC development and progression remain to be explored further.

In our research, we demonstrated that hsa‐circ‐0052001 promoted the tumorigenesis of GC. The expression of hsa‐circ‐0052001 is overexpressed in GC tissues. Mechanistically, hsa‐circ‐0052001 promotes the proliferation and metastasis of GC cells via the MAPK pathway. Thus, hsa‐circ‐0052001 and the MAPK pathway are a potential target for the diagnosis and treatment of GC patients.

## 2 MATERIALS AND METHODS

### 2.1 Gastric cancer tissues and ethical approval

Surgical excision of GC patients without preoperative chemoradiotherapy at The First Affiliated Hospital of Soochow University acquired 102 GC tissues and adjacent noncancerous specimens between 2015 and 2017. Following surgical resection, all samples were collected and placed into liquid nitrogen until RNA extraction. Two pathologists independently verified all clinicopathological diagnoses. The First Affiliated Hospital of Soochow University Ethics Committee authorized the trial, and each patient gave written informed permission before the operation (IRB number: 2021112).

### 2.2 Bioinformatics analysis

CircRNAs expression profile (GSE83521) was downloaded from the NCBI GEO database (https://www.ncbi.nlm.nih.gov/geo/), comprising data for 6 GC and 6 paracancer tissues. Differently expressed genes (DEGs) between GC and paracancer tissues were identified from an online database (https://www.ncbi.nlm.nih.gov/geo/geo2r/) based on |logFC| > 1 and adj. *p*‐value < 0.05. The Benjamini and Hochberg false discovery rate (FDR) approach was utilized to explain the emergence of FDR via the modified *P*‐values. MiRNAs that might directly bind to circRNAs were predicted using data in the CircBank database (https://www.circbank.cn). The gene targets for the predicted miRNAs were identified in the TargetScan database. The cumulative weight context++ score < −0.6 were set as the cutoff. The function of the circRNAs and downstream target genes was investigated further using Gene Ontology (GO)^13^ and Kyoto Encyclopedia of Genes and Genomes (KEGG)^14^ analysis. Subsequently, GO and KEGG enrichment analysis were conducted using the clusterProfiler R package, as previously described.^15^

### 2.3 Cell culture

Human GC cell lines (AGS, MKN‐45, SGC7901) and normal gastric mucosal epithelial cell line (GES‐1) were provided by the Culture Collection of the Chinese Academy of Sciences (Shanghai, China). At 37°C under 5% CO2, AGS, MKN‐45, and SGC7901 cells were cultivated in RPMI 1640 medium (Hyclone), whereas GES‐1 cell was cultivated in DMEM medium (Hyclone) added containing 10% FBS (BI, Israel) and a mixture of 1% penicillin/kyowamycin (Gibco, America).

### 2.4 Total RNA isolation and qRT‐PCR

Following the manufacturer's instructions, RNA was extracted from GC cells and tissues via TRIZOL reagent (Thermo Fisher Scientific, America). Substantial, Nano Drop 2000 (Thermo Scientific, America) was used to identify the concentration and purity of the RNA. For circular RNA and mRNA, reverse transcription was conducted with random hexamers using HiScriptIIQ Select RT Super Mix for qPCR (+gDNA wiper) (Vazyme, China). For miRNA, cDNA was synthesized from the miRNA 1st Strand (Vazyme, China) using the Stem‐loop primer. TB Green Premix Ex Taq II (Takara, Japan) was used to amplify cDNA using an ABI Prism 7500 (Applied Biosystems, USA). Internal controls for circRNA and mRNA were GAPDH, and all samples were run three times in total. The expression of genes was calculated using the 2^−ΔΔCT^ method was utilized. The primers utilized herein are presented in Additional file 1: Table S1.

### 2.5 Transfection

GC cells were planted and cultured until 80% confluence in 6‐well plates before transfection. GenePharma (Shanghai, China) developed and manufactured si‐circ‐0052001, miR‐608 inhibitor, and their representative negative control oligonucleotides. Lipofectamine^TM^ 2000 (Invitrogen, America) was utilized to realize the transfection of the oligonucleotides and plasmids into cells following the manufacturer's protocol. The oligonucleotide sequences are shown in Table S2.

### 2.6 The CCK‐8 assay

We seeded the transfected AGS and MKN45 cells in a 96‐well dish at 3000 cells per well. After 24, 48, 72, and 96 h of incubation, 10 μl of CCK‐8 liquor (Vazyme, China) were added to the wells. The absorbance of the wells at 450 nm was measured with a full‐wavelength marker after 2 h of incubation at 37°C.

### 2.7 Colony formation assay

The treated GC cells were seeded in 6‐well dishes and cultured under 37°C for 2 weeks with 500 cells in each well. We fixed the cells in 4% PFA and stained the cells in gentian violet (Beyotime, China) for 30 min, followed by two washes in PBS. After that, a count and evaluation of colonies were conducted.

### 2.8 Wound‐healing and Transwell assays

We planted the transfected GC cells in a 6‐well plate. A scratch was made from the top to the center at the bottom of the well. Cells were washed twice in PBS and cultivated in the RPMI 1640 medium without serum under 37°C for 24 h. After that, at 0 h and 24 h, image collection and migration distance measurement were performed.

The Transwell chamber was put in a 24‐well plate for transwell analysis. The treated MKN45 and AGS cells in the medium without serum were seeded into the upper layer, and 700 μl of complete medium were supplemented into the lower layer. Posterior to the 24h cultivation, cells passing through the film were fixed in 4% PFA and stained in gentian violet for 15 min. Count the number of invasive GC cells, then repeat the experiment three times.

### 2.9 Western blot

In order to identify the concentration of proteins in GC cells expressing si‐circ‐0052001, RIPA buffer (Epizyme, China) was prepared and the protein content was identified using the BCA method (Beyotime, PRC). Protein extracted from GC cells was separated on SDS‐PAGE and transferred onto PVDF membranes. Non‐specific binding sites were blocked from PVDF membranes using 5% skimmed milk under ambient temperature for 1.5 h. The cultivation with the first antibody was completed overnight under 4°C. Subsequently, cultivation with the second antibody was completed at room temperature for 1 h. The protein band images were captured using Odyssey and analyzed using the ImageJ software. Additional file 1: Table S3 lists the antibodies utilized in our research.

### 2.10 Xenografts in mice

The Animal Ethics Committee at Soochow University approved animal care and experiments (Suzhou, China). Male SPF BALB/c nude mice for tumor heterograft assays were obtained from Shanghai SLRC Lab Animal Company. The mice were about 4–5 weeks old and weighed 15–16 g. The experiment started after 3 days of acclimatization. On the day of the experiment, the mice were randomly divided into the hsa‐circ‐0052001 knockdown or control groups, each containing six mice. Mice in the treatment group received 5 × 10^6^ of si‐circ cells, whereas than in the control group received an injection of si‐NC MKN45 cells into the left or right dorsal flanks.

### 2.11 Statistics

SPSS 23.0 software (IBM, America), GraphPad Prism 7, and R programs were employed to analyze the data. Continuous normally distributed data were expressed as mean and SD. The expressing level of hsa‐circ‐0052001 in GC and adjacent noncancerous specimens were contrasted via a paired *t* test. The connection among hsa‐circ‐0052001 expressing level and clinicopathologic characteristics were investigated via the Chi‐square test. In contrast among the other two groups of data, an independently completed sample t test was used. *p <* 0.05 was considered statistically significant.

## 3 RESULTS

### 3.1 Hsa‐circ‐0052001 was determined as a GC‐related circRNA

**FIGURE 1:**
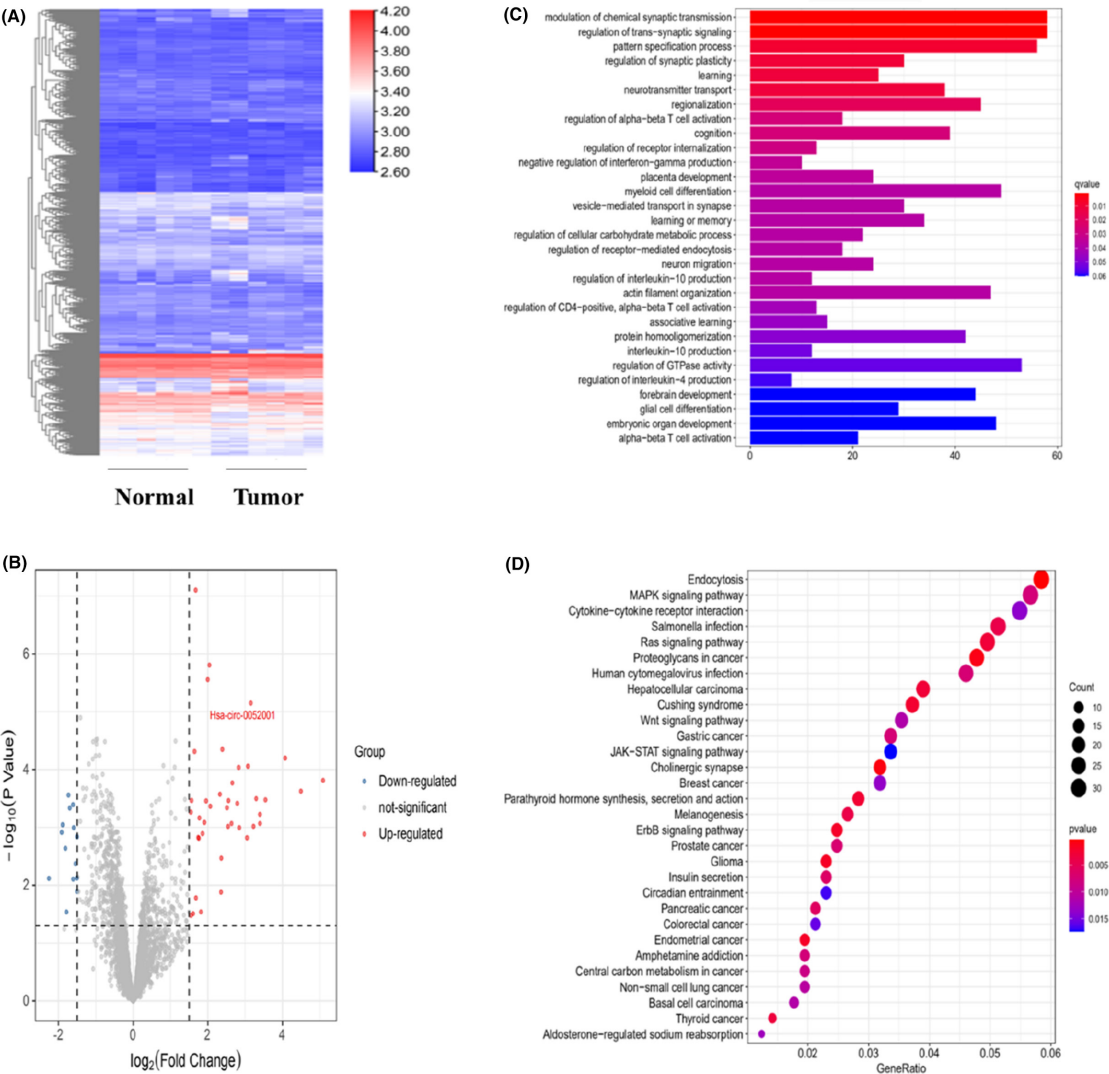
The analysis of the GSE83521 database and the function of hsa‐circ‐0052001 in GC. (A) A heatmap of all circRNAs in GSE83521 dataset. (B) A volcano map of the differentially expressed circRNAs in GSE83521 dataset. (C‐D) GO and KEGG pathway of hsa‐circ‐0052001.

The GSE83521 dataset in the GEO database contains data for circRNAs in GC tissues. We found 40 circRNAs highly expressed in GC tissues and 12 underexpressed circRNAs (Figure 1A,B). Hsa‐circ‐0052001 was found to be considerably overexpressed in GC tissues relative to paracancerous samples. Further search in the CircBank database revealed that miR‐1184, miR‐608, miR‐6737‐5p, miR‐6742‐5p, miR‐6768‐5p, and miR‐6812‐5p are the possible targets of hsa‐circ‐0052001, and then, the online database TargetScan was employed to forecast the mRNAs regulated by the above miRNAs, the cumulative weight context++ score < −0.6 were set as the cutoff. The circRNA‐miRNA‐mRNA network performed using Cytoscape is shown in Figure S1. GO analysis revealed that hsa‐circ‐0052001 mainly regulates the secretion of interleukin‐10 and the GTPase activity (Figure 1C). KEGG analysis suggested that the DEGs were primarily associated with the following functions: MAPK signaling pathway and gastric cancer (Figure 1D).

### 3.2 Hsa‐circ‐0052001 is overexpressed in GC tissues and predicted a worse prognosis

**FIGURE 2:**
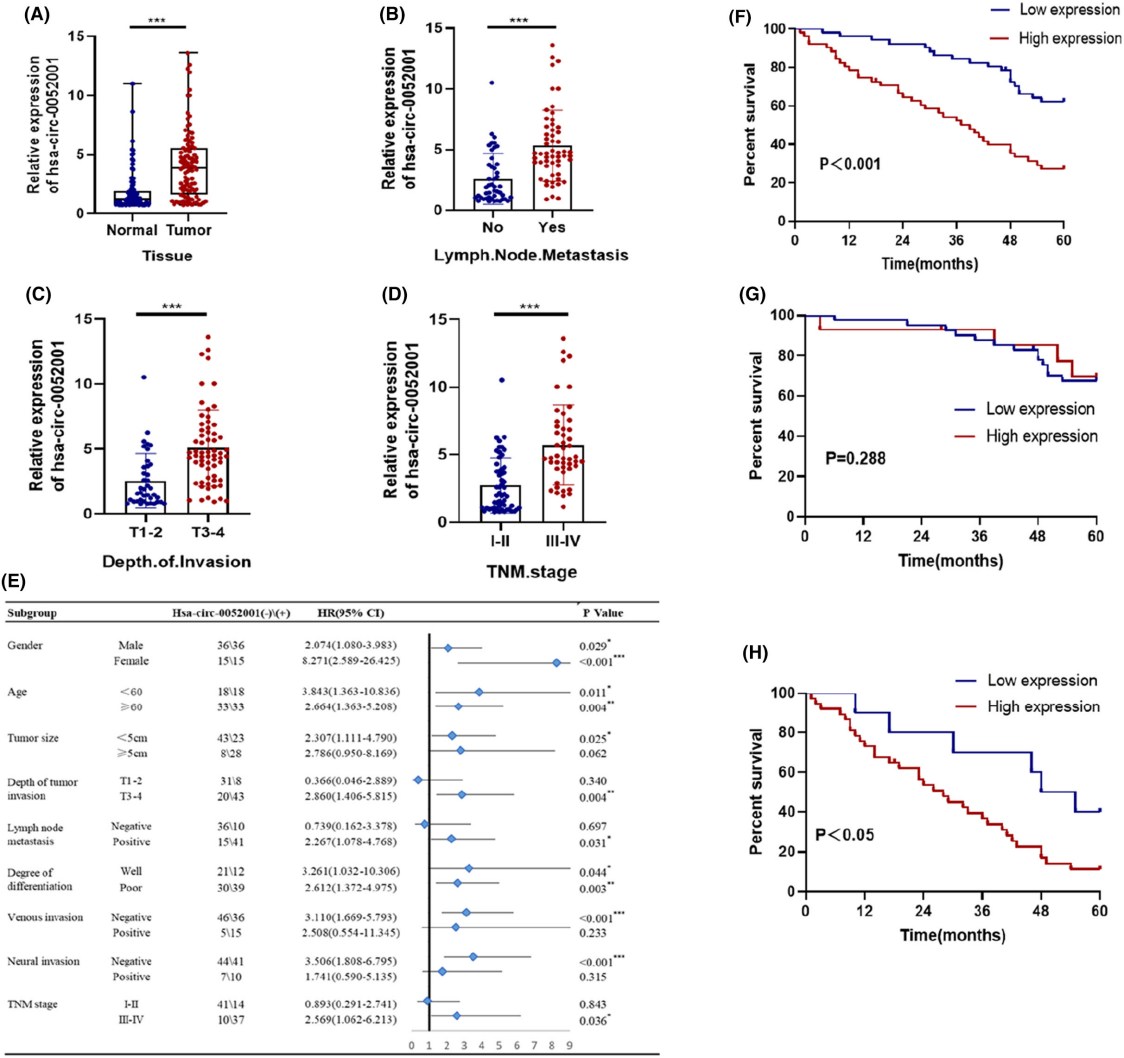
Expression of hsa‐circ‐0052001 in GC tissues. (A‐D) Expression levels of hsa‐circ‐0052001 in (A) GC vs normal tissues, (B) tumors with or without lymph node invasion, (C) T I‐II vs T III‐IV tissues, and (D) early vs late TNM staging. E OS of hsa‐circ‐0052001pos and hsa‐circ‐0052001neg GC patients in subgroups demarcated by age, gender, tumor size, depth of tumor invasion, lymph node metastasis, degree of differentiation, vascular invasion, nerve invasion, and TNM staging. F‐H OS of (F) hsa‐circ‐0052001^pos^ and hsa‐circ‐0052001^neg^ GC patients with TNM staging I‐II (G) and III‐IV (H). *p < 0.05, **p < 0.01, ***p < 0.001.

To further confirm the overexpression of hsa‐circ‐0052001 in GC tissues, we assessed the expression of this circRNA in 102 GC specimens. The results confirmed that hsa‐circ‐0052001 expression was considerably high in GC tissues than in normal adjacent noncancerous tissues, consistent with bioinformatic analysis findings (Figure 2A). Furthermore, hsa‐circ‐0052001 expression was overexpressed in patients who had lymph node invasion than in those without (Figure 2B) and upregulated in the T3‐4 stage compared with the T1‐2 stage (Figure 2C). We also found that the hsa‐circ‐0052001 were upregulated in TNM phase III‐IV patients vs I‐III (Figure 2D). In addition, when we analyzed the clinicopathological feature of the GC patients, we discovered that hsa‐circ‐0052001 expression level was evidently related to tumor size, T grade, lymph node invasion, TNM stage, and vascular invasion (Table 1), but there was no obvious association with other clinicopathologic characteristics such as age, sex, histological grade, or nerve invasiveness (Table 1). Based on differences in hsa‐circ‐0052001 expression levels, patients are divided into two groups. Patients with lower hsa‐circ‐0052001expressing level had a considerably longer OS in contrast to patients with higher expressing level, according to our findings (Figure 2E,F). Interestingly, a high level of hsa‐circ‐0052001 was linked to poor prognoses in TNM phase III‐IV patients, but not in phase I‐II patients (Figure 2G,H). These findings could imply that hsa‐circ‐0052001 was a significant prognosis marker, especially for those with advanced gastric cancer.

**TABLE 1:** Relationship between hsa‐circ‐0052001 and clinic‐pathological factors in 102 GC patients

Variables	Hsa‐circ‐0052001		
	Negative	Positive	*p* value
Age (years)			
<60	18	18	1.000
≥60	33	33	
Gender			
Male	36	36	1.000
Female	15	15	
Tumor size (cm)			
<5	43	23	<0.001***
≥5	8	28	
T grade			
T1‐2	31	8	<0.001***
T3‐4	20	43	
N grade			
N0	36	10	<0.001***
N1‐3	15	41	
Histological grade			
Middle‐High	21	12	0.057
Low	30	39	
Vascular invasion			
Negative	46	36	0.013*
Positive	5	15	
Neural invasion			
Negative	44	41	0.425
Positive	7	10	
TNM staging			
I–II	41	14	<0.001***
III–IV	10	37	

* *p* < 0.05.

*** *p* < 0.001.

### 3.3 Hsa‐circ‐0052001 promotes the proliferation, migration, and invasion of GC cells in vitro

**FIGURE 3:**
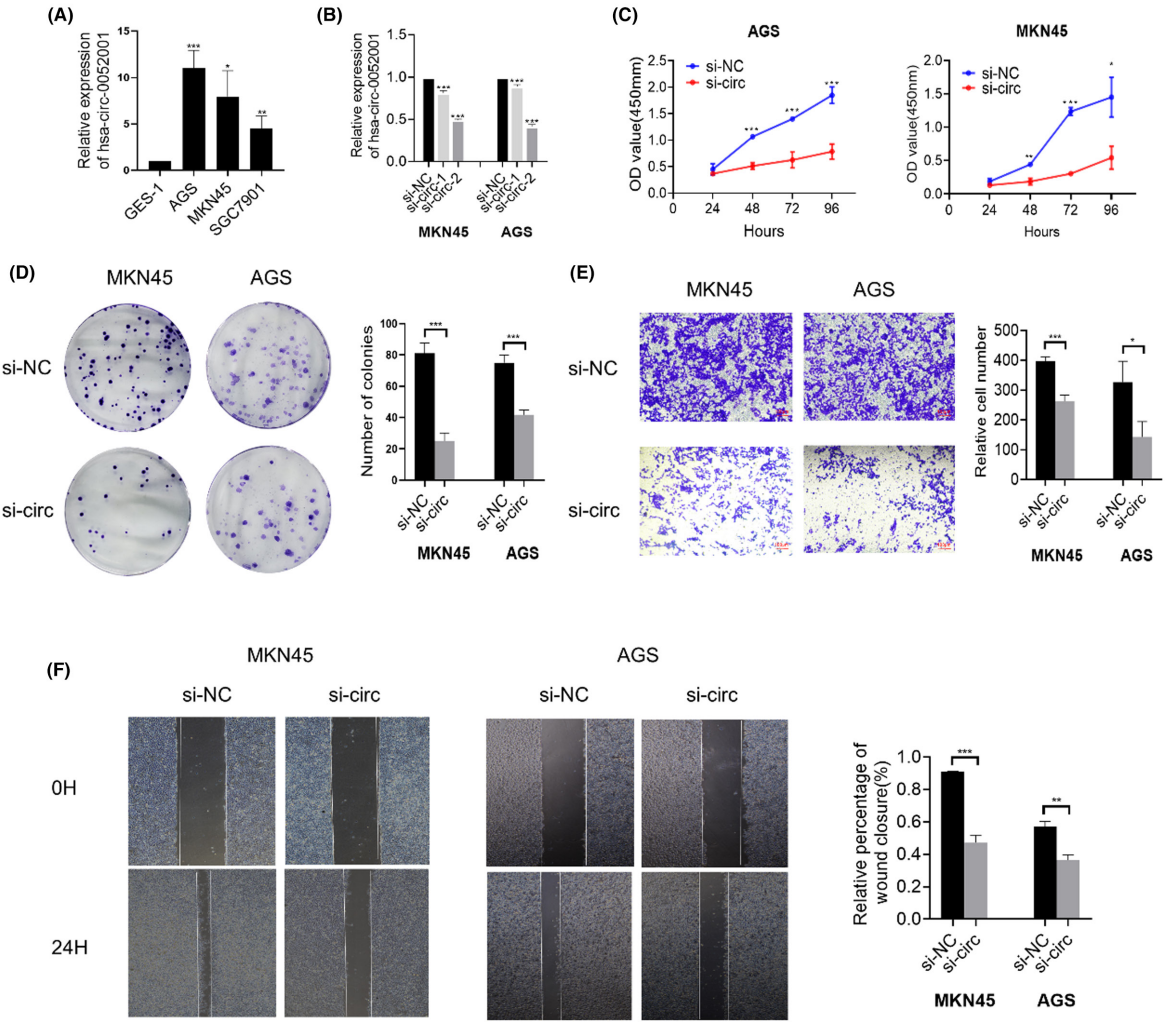
The effect of hsa‐circ‐0052001 knocking down on the proliferation, migration, and invasion of GC cells. (A) The expression level of hsa‐circ‐0052001 in GC cells and GES‐1 cell lines was measured using qPCR. (B) qPCR analysis for the expression of hsa‐circ‐0052001 in MKN‐45 and AGS cells transfected with siRNA. (C) CCK‐8 analysis for the effect of hsa‐circ‐0052001 on the proliferation of MKN45 and AGS cells. (D) Colony‐formation analysis for the effect of hsa‐circ‐0052001 on the colony‐forming abilities of MKN45 and AGS cells. (E) Transwell assay for the effect of hsa‐circ‐0052001 on the invasiveness of MKN45 and AGS cells. (F). Wound healing analysis for the effects of hsa‐circ‐0052001 on the migration of MKN45 and AGS cells. *p < 0.05, **p < 0.01, ***p < 0.001.

Our preliminary study demonstrated that hsa‐circ‐0052001 is overexpressed in GC tissues than in normal gastric epithelium tissues. Further in vitro studies were performed in AGS and MKN45 cells (Figure 3A). The role of hsa‐circ‐0052001 on the biological behavior of GC cells was assessed using loss‐of‐function assays. First, different siRNAs that target the hsa‐circ‐0052001 back‐splice area were designed. After transfection of the siRNAs to AGS and MKN45 cells, hsa‐circ‐0052001 was successfully knocked down (Figure 3B). Finally, si‐circ0052001‐2 was selected for the next experiments because of its high inhibitory efficiency. CCK‐8 experiment revealed that down‐regulating hsa‐circ‐0052001 expression inhibited the growth of MKN45 and AGS cells (Figure 3C). Also, downregulating hsa‐circ‐0052001 inhibited the colony formation property of GC cells (Figure 3D). Wound repair and Transwell analysis were utilized to identify the influences of hsa‐circ‐0052001 on GC cell metastasis and invasion. Our assays revealed that downregulating hsa‐circ‐0052001 expressively suppresses the metastasis and invasion of MKN45 and AGS cells (Figure 3E,F). Generally, knocking down hsa‐circ‐0052001 expression inhibited the proliferation, migration, and invasion of GC cells.

### 3.4 MiR‐608 inhibitor inhibited the effect of hsa‐circ‐0052001

**FIGURE 4:**
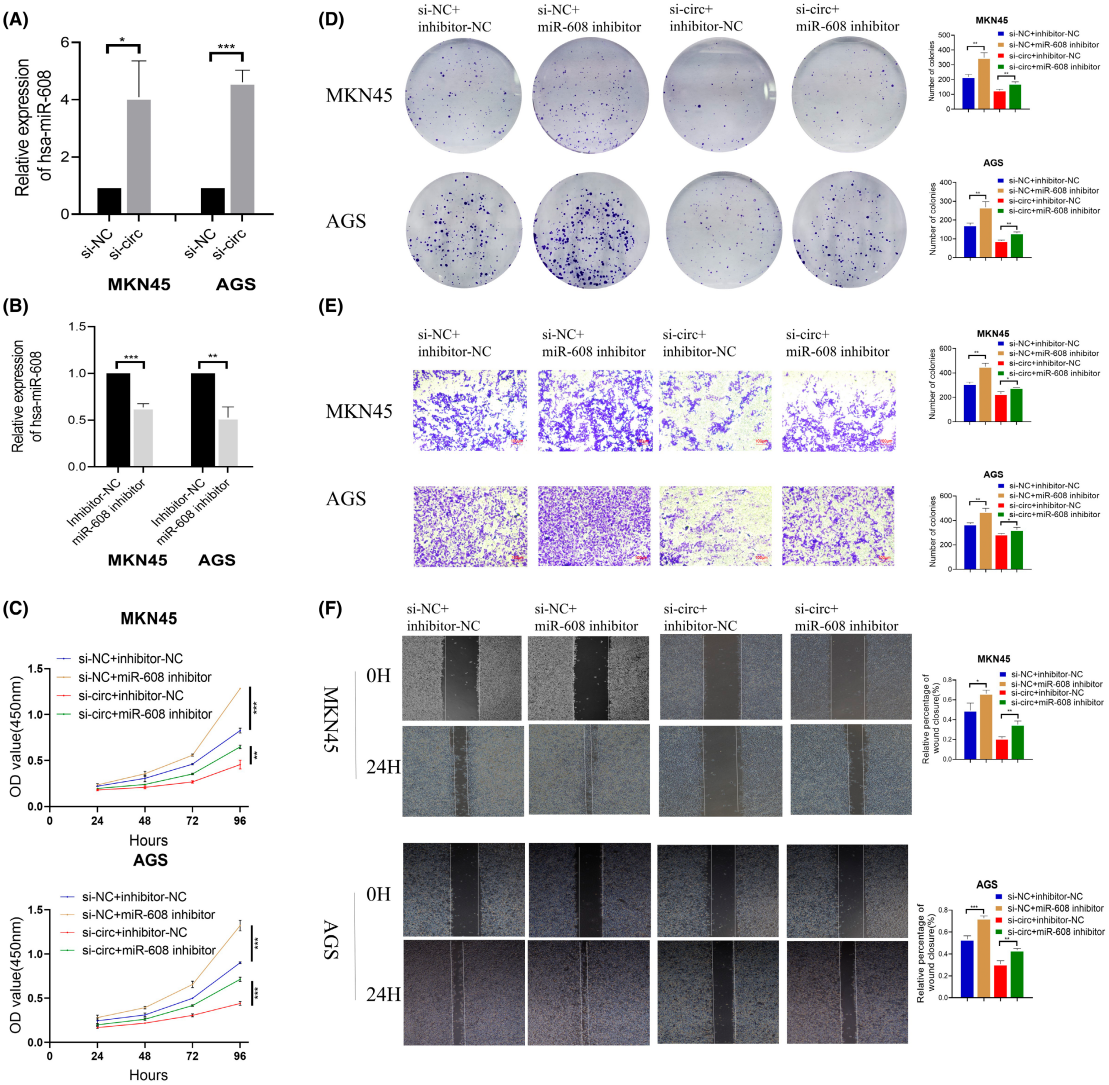
The effect of MiR‐608 on the inhibitory effect of hsa‐circ‐0052001 on the proliferation, migration, and invasion of GC cells. (A) The expression of MiR‐608 in hsa‐circ‐0052001 knock down GC cells. (B) qPCR analysis for the efficacy of miR‐608 expression inhibition in MKN45 and AGS cells. (C‐F) CCK‐8, Colony formation analysis, Wound‐healing assay, and Transwell analysis for the effect of miR‐608 inhibition on the proliferation, migration, and invasion of MKN45 and AGS cells. *p < 0.05, **p < 0.01, ***p < 0.001.

We found when hsa‐circ‐0052001 was knocked down, miR‐608 expressing level was relatively upregulated in GC cells (Figure 4A). As a result, we hypothesized that hsa‐circ‐0052001 promoted the proliferation and invasion of GC cells via binding to miR‐608 in a competitive manner. MiR‐608 suppressor was transfected into GC cells via transfection and verified its downregulation efficiency (Figure 4B). The CCK‐8 and colony formation assays implied that the miR‐608 inhibitor rescued the impact of hsa‐circ‐0052001 on MKN45 and AGS cell proliferation (Figure 4C,D). In addition, the downregulation of miR‐608 expression reversed the inhibitory impact of hsa‐circ‐0052001 downregulation on MKN45 and AGS cell migration and invasion (Figure 4E,F). The discoveries herein showed that hsa‐circ‐0052001 promoted the development of GC cells through binding with miR‐608.

### 3.5 Hsa‐circ‐0052001 promotes the proliferative, migratory, and invasion of GC cells via the MAPK signaling pathway

**FIGURE 5:**
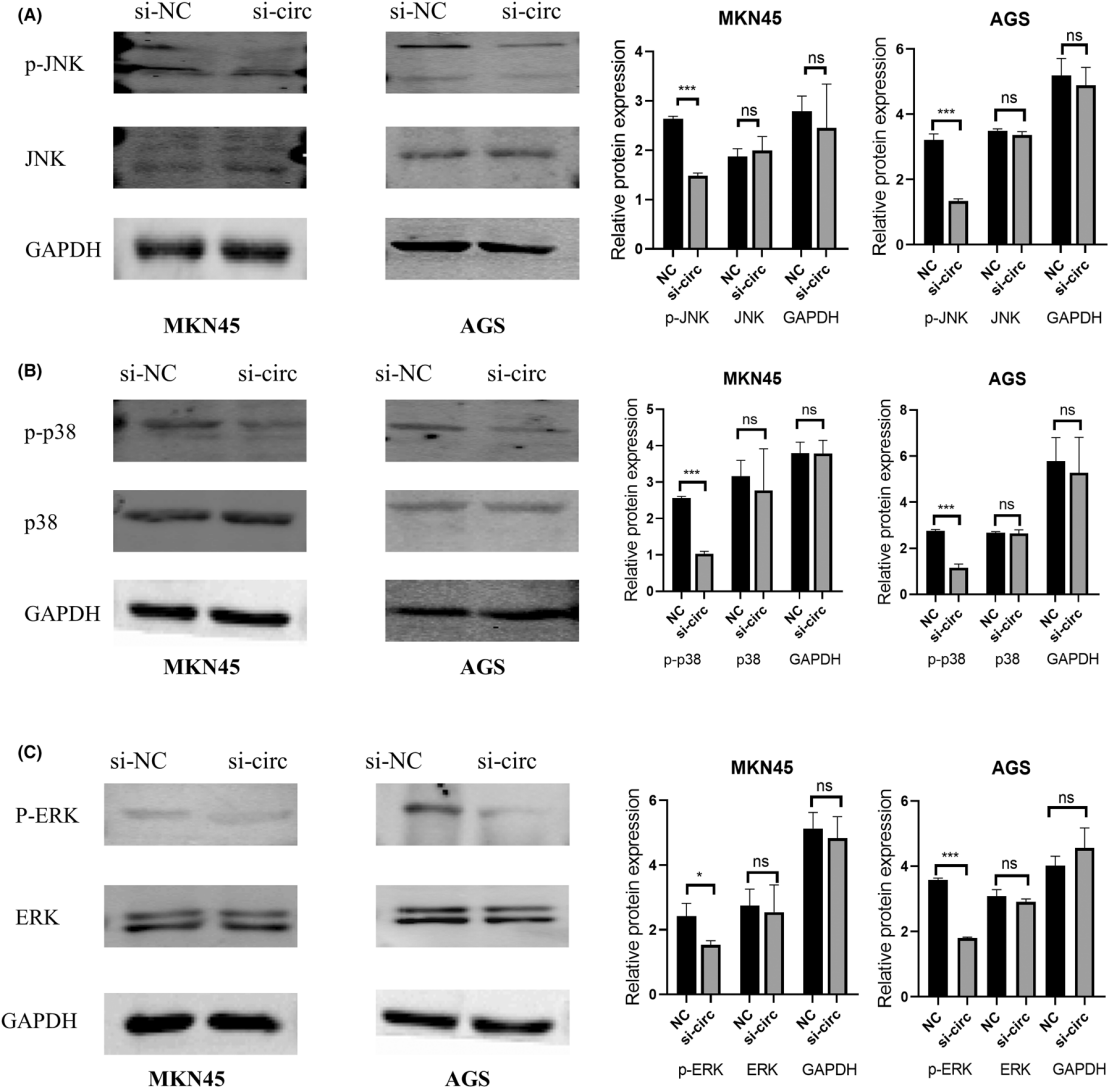
Hsa‐circ‐0052001 active the ERK/MAPK pathway. (A) The expression of JNK, p‐JNK, and GAPDH in GC cells. (B) The expression of p38, p‐p38, and GAPDH in GC cells. (C) Western blot for the effect of downregulating hsa‐circ‐0052001expression on the expression of ERK, p‐ERK, and GAPDH in GC cells.

Previous research have demonstrated that miR‐608 can activate the MAPK pathway in ameloblastoma.^16^ Therefore, we hypothesized whether hsa‐circ‐0052001 regulates the MAPK pathway through miR‐608 in gastric cancer. Western blotting was used to analyze key proteins connected to tumor proliferation and metastasis in the MAPK signaling pathway. The findings demonstrated that p‐ERK, p‐P38, and p‐JNK were considerably reduced in hsa‐circ‐0052001 knockdown GC cells, but there was no significant differences in ERK, P38, or JNK in knockdown cells (Figure 5A‐C). These findings proved that the downregulation of hsa‐circ‐0052001 could suppress the MAPK pathway.

### 3.6 Hsa‐circ‐0052001 promotes the growth of gastric cancer cells in vivo

**FIGURE 6:**
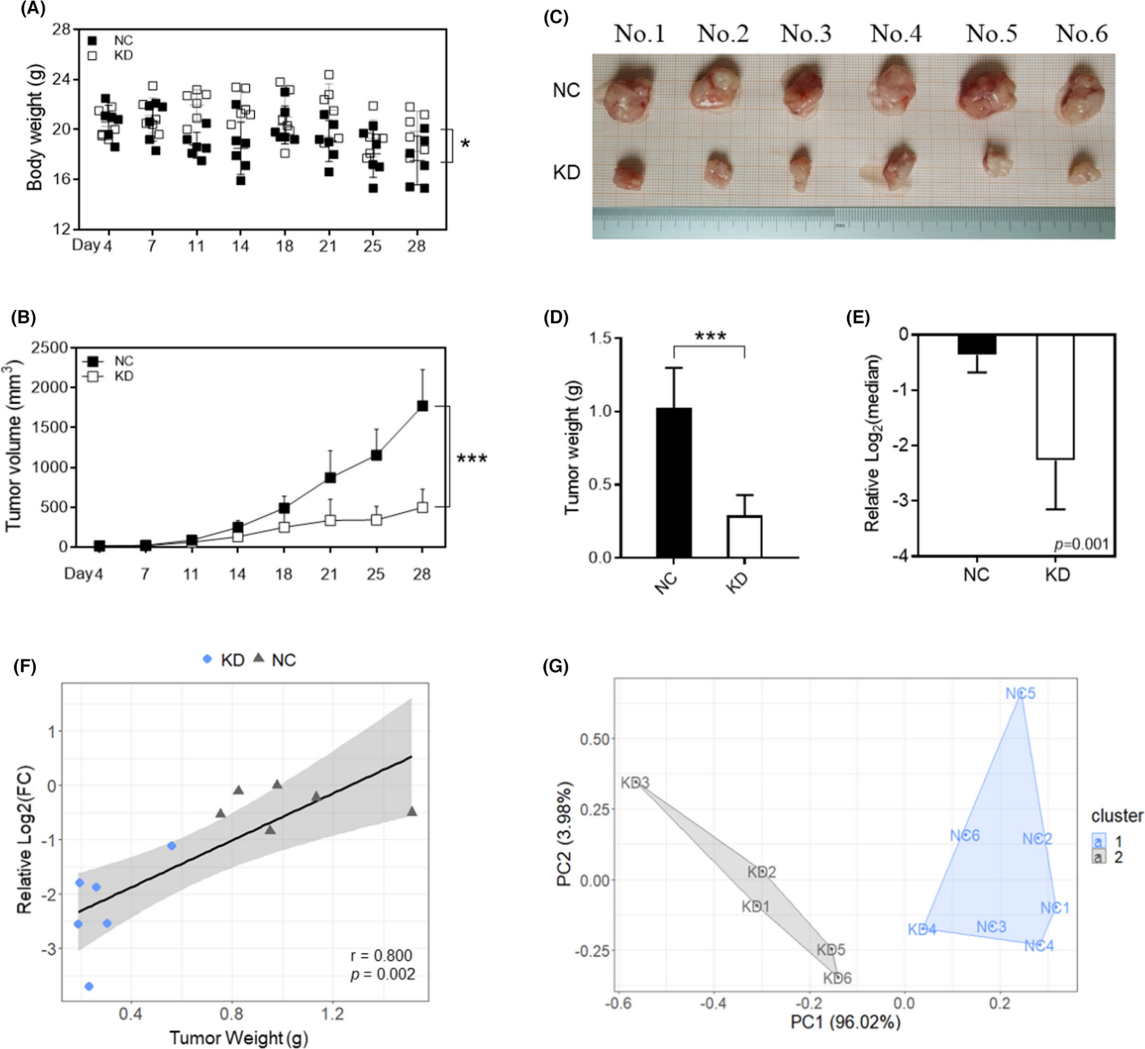
The effect of hsa‐circ‐0052001 knockdown on the growth of GC cells in vivo. A‐B Analysis of bodyweight (A) and volume of tumor (B). (C) Image of subcutaneous tumor tissues in the NC group and the hsa‐circ‐0052001‐KD group. (D) The tumor weight. (E) Relative hsa‐circ‐0052001 levels in the tumors and its (F) relevance with tumor weight. (G) Mice were divided into groups 1 (gray) and 2 (blue) based on tumor volume, body weight, tumor weight, and hsa‐circ‐0052001 levels.

The mouse GC model was established to further assess the impact of hsa‐circ‐0052001 on the GC growth in vivo. MKN45 cells, which were transfected with si‐NC or si‐circ‐0052001, were injected subcutaneously into each mice. Compared with mice in the NC group, inhibiting hsa‐circ‐0052001 expression had no significant effects on the bodyweight of the mice (Figure 6A), but remarkably reduced the tumor size (Figure 6B,C) and weight (Figure 6D). Furthermore, hsa‐circ‐0052001 levels were markedly lower in the tumors in mice injected with si‐circ‐0052001 (Figure 6E) and showed a positively correlated with tumor weight (Figure 6F). Then, a cluster analysis was performed combining the effects of tumor volume, body weight, tumor weight, and hsa‐circ‐0052001 level (Figure 6G). These findings suggested that downregulation of hsa‐circ‐0052001 may suppress tumor growth in vivo.

## 4 DISCUSSION

The most common malignant tumor of the digestive tract is Gastric Cancer. Due to difficulty in early GC diagnosis, the prognosis of gastric cancer patients is usually poor. The molecular mechanism underlying the development and progression of gastric cancer is still unclear. To improve the OS of GC patients, there is a need to develop accurate markers for early‐stage GC diagnosis. CircRNAs have emerged as hot spots for cancer research, thanks to the recent advances in RNA sequencing technology and bioinformatics analysis.^17^ Some circRNAs have been revealed to be oncogenic genes or cancer inhibitor genes in lots of tumors, such as lung carcinoma, colon carcinoma, and hepatocellular carcinoma.^18^, ^19^, ^20^ However, the connection between circRNAs and GC is unclear, and the specific mechanism underlying the effect of circRNAs in GC remains to be validated. In the present study, we explored how hsa‐circ‐0052001 promotes GC progression.

In our research, bioinformatics analysis first revealed that hsa‐circ‐0052001 is overexpressed in GC cells and tissues. Further analyses revealed that hsa‐circ‐0052001 was linked to tumor size, T grade, lymphonodus invasion, TNM phase, vascular invasion, and GC prognosis. Studies show that hsa‐circ‐0052001 promotes the proliferative and invasion of GC cells in vitro by binding to hsa‐miR‐608 and could activate the MAPK pathway. Accordingly, hsa‐circ‐0052001 is a potential target for GC diagnosis, treatment, and prognosis prediction.

CircRNAs regulate cancer progression by acting as ceRNAs.^21^, ^22^, ^23^, ^24^ In this study, CircBank analysis predicted that hsa‐circ‐0052001 has binding sites for hsa‐miR‐608. Thus, rescue experiments confirmed that hsa‐circ‐0052001 reversed the tumor suppressor roles of miR‐608. Other circRNAs also participate in GC development by acting as ceRNA. For example, overexpression of circDLST promoted the metastasis and progression of GC through binding to miR‐502‐5p.^25^ Moreover, circCACTIN plays the role of miR‐331–3p sponge, and circCACTIN promotes GC progression by upregulating the expression of TGFBR1.^26^ Furthermore, circDLG1 upregulated the expression of CXCL12 via binding to miR‐141–3p, which promotes GC progression and resistance to PD‐1.^27^ Our study revealed that hsa‐circ‐0052001 promoted the proliferation and invasion of GC by sponging hsa‐miR‐608. Hsa‐miR‐608, a cancer inhibitor, was discovered to take part in diverse tumors. A report indicated that miR‐608 could downregulate EGFR and p53, which might closely relate to the progression of cancer via the MAPK signaling pathway.^16^ Another report claimed that lncRNA BLACAT1 promoted osteosarcoma carcinoma development through downregulating miR‐608 and increasing the expression of SOX12.^28^ Also, miR‐608 downregulates the expression of TFAP4 to promote Doxorubicin‐induced apoptosis in NSCLC tissue.^29^

In the present study, bioinformatics analysis revealed that hsa‐circ‐0052001 may be related to gastric cancer and the MAPK pathway. Western blot was used to analyze key proteins related to tumor proliferation and invasion in the MAPK signaling pathway. The results showed the suppression of hsa‐circ‐0052001 downregulated p‐ERK, p‐P38, and p‐JNK, while there was no significant change in ERK, P38, and JNK, further confirming that hsa‐circ‐0052001 could promote the proliferative and invasion of GC cells via the MAPK pathway. MAPK pathway is a key signaling pathway involved in diverse cancers and other human diseases.^30^, ^31^, ^32^ Numerous studies have linked circRNAs to the MAPK pathway. For example, hsa‐circ‐0003204 promotes cervical cancer tumorigenesis by inhibiting MAPK signaling pathway.^33^ Circ0001313 remarkably strengthened NSCLC cell proliferation and invasion by enhancing the MAPK pathway.^34^

As far as we know, this is the first report on the expression, role, relationship with clinical features, and the mechanism underlying the effect of hsa‐circ‐0052001 on GC cells. Generally, we found that hsa‐circ‐0052001 is highly expressed in gastric cancer tissues, and its expression is related to the prognosis of gastric cancer patients. This indicates that hsa‐circ‐0052001 is a potential marker for the treatment, diagnosis, and prognosis prediction of GC. Regarding limitations, our preliminary study first shows that hsa‐circ‐0052001 expression is upregulated in GC cells and tissues. However, whether it is also overexpressed in the blood is unknown. Second, our research demonstrated that hsa‐circ‐0052001 could activate the MAPK pathway, but the specific mechanism is still unclear, and needs further investigation. Third, in addition to binding to miR‐608, other hsa‐circ‐0052001 target miRNAs which may impact the progression and developmental process of GC are unknown. Therefore, other aspects of hsa‐circ‐0052001 in relation to GC need further investigation.

## 5 CONCLUSION

Summarily, this study revealed that hsa‐circ‐0052001 is highly expressed in gastric cancer tissues and is closely related to the prognosis of gastric cancer. Hsa‐circ‐0052001 promotes GC development and progression by binding to the miR‐608. Moreover, our data revealed that hsa‐circ‐0052001 could promote the MAPK pathway and promote the growth of GC cells in vivo. Thus, hsa‐circ‐0052001 is not only a prognostic marker for gastric cancer patients but also a diagnostic and prognosis prediction for GC.

## AUTHOR CONTRIBUTIONS

**Qixuan Xu:** Data curation (equal); methodology (equal); software (equal); writing – original draft (equal). **Yizhou Yao:** Methodology (equal); software (equal). **Haishun Ni:** Methodology (equal). **Jinrong Gu:** Project administration (equal); resources (equal). **Xuchao Wang:** Formal analysis (equal). **Linhua Jiang:** Resources (equal); validation (equal). **Bin Wang:** Software (equal); visualization (equal). **Xinguo Zhu:** Conceptualization (equal); supervision (equal); writing – review and editing (equal).

## CONFLICT OF INTEREST

There are no conflicts of interest according to the authors.

## ETHICS APPROVAL AND CONSENT TO PARTICIPATE

The First Affiliated Hospital of Soochow University's Ethics Review Committee authorized the study (Suzhou, China). The animal study followed the First Affiliated Hospital of Soochow University's Guidelines for Animal Care and Use.

## Supporting information


Table S1.
Table S2.
Table S3.

Figure S1.

## Data Availability

The datasets used and/or analyzed during the current study are available from the corresponding author on reasonable request.
