# Supplementary material for: Hsa‐circ‐0052001 promotes gastric cancer cell proliferation and invasion via the MAPK pathway
Source: Cancer Med. 2022 Dec 1;12(6):7246–57. doi: 10.1002/cam4.5446 (PMC10067131; doi:10.1002/cam4.5446)
Supplement: Supplementary file 1 — Table S1. Table S2. Table S3. [file CAM4-12-7246-s002.doc]

| Name | Primer | Sequence |
| --- | --- | --- |
| Hsa-circ-0052001 | Forward primer | TCATCAACCCGTACAAGCAGCTTC |
|  | Reverse primer | GCACCTCGTGGCGCTTCTTG |
| GAPDH | Forward primer | CAGGAGGCATTGCTGATGAT |
|  | Reverse primer | GAAGGCTGGGGCTCATTT |
| miR-608 | Forward primer | GGGGTGGTGTTGGGACAG |
|  | Reverse primer | AGTGCAGGGTCCGAGGTATT |
|  | Stem-loop primer | GTCGTATCCAGTGCAGGGTCCGAGGTATTCGCA  CTGGATACGACACGGAG |

**Table S1** Primers used in this study

**Table S2 Oligonucleotides used in this study**

| Definition | Sequence(5’-3’) |
| --- | --- |
| si-hsa-circ-0052001-1 | CUGCAGGACGUACUCCGGCTT  GCCGGAGUACGUCCUGCAGTT |
| si-hsa-circ-0052001-2 | GCAUGCUGCAGGACGUACUTT  AGUACGUCCUGCAGCAUGCTT |
| si-hsa-circ-0052001-NC | ACGUGACACGUUCGGAGAATT |
| hsa-miR-608 inhibitor | ACGGAGCUGUCCCAACACCACCCCU |
| hsa-miR-608 NC | CAGUACUUUUGUGUAGUACAA |

Table S3 Antibodies used in this study

| Antigens | Manufacturers | Applications |
| --- | --- | --- |
| p-JNK | AF3318, Affinity | 1:1000 for WB |
| JNK | bs-2592R, Bioss | 1:1000 for WB |
| p-p38 | bs-0636R, Bioss | 1:1000 for WB |
| P38 | bs-0637R, Bioss | 1:1000 for WB |
| p-ERK | bs-3016R, Bioss | 1:1000 for WB |
| ERK | bsm-33337M, Bioss | 1:1000 for WB |
| GAPDH | 10494-1-AP, Proteintech | 1:5000 for WB |
