# Supplementary material for: Hsa‐circ‐0052001 promotes gastric cancer cell proliferation and invasion via the MAPK pathway
Source: Cancer Med. 2022 Dec 1;12(6):7246–57. doi: 10.1002/cam4.5446 (PMC10067131; doi:10.1002/cam4.5446)

**Figure S1** The circRNA-miRNA-mRNA network of hsa-circ-0052001. The red rhombus represents hsa-circ-0052001, the blue square represent miRNAs, and the green round represent mRNAs.


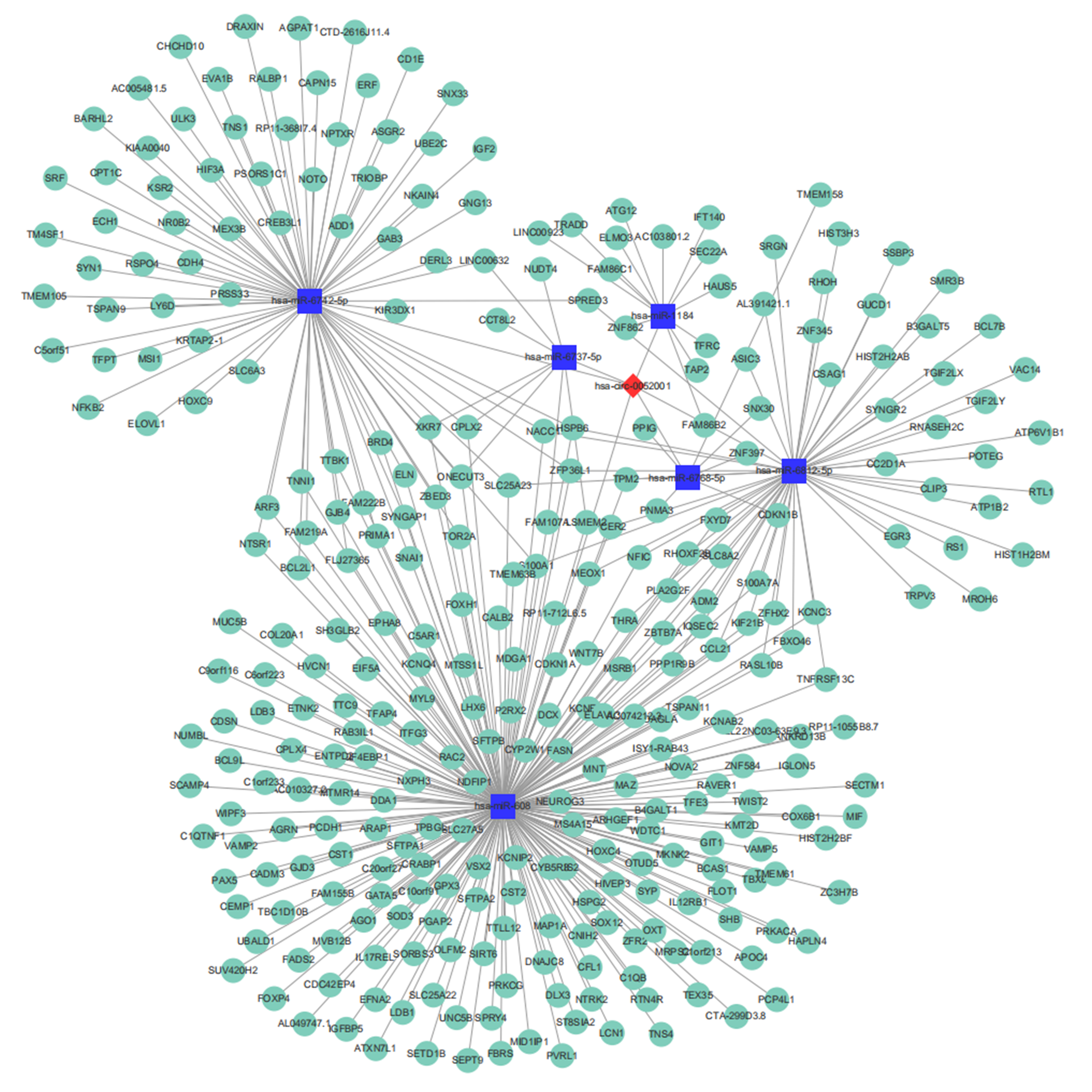

Supplement: Supplementary file 2 — Figure S1. [file CAM4-12-7246-s001.doc]
